# Acupuncture for the treatment of urinary incontinence: A review of randomized controlled trials

**DOI:** 10.3892/etm.2013.1210

**Published:** 2013-07-09

**Authors:** SUN-HO PAIK, SU-RYUN HAN, OH-JUN KWON, YOUNG-MIN AHN, BYUNG-CHEOL LEE, SE-YOUNG AHN

**Affiliations:** Department of Internal Medicine, College of Korean Medicine, Kyung Hee University, Dongdaemun-gu, Seoul 130-702, Republic of Korea

**Keywords:** acupuncture, acupressure, urinary incontinence, leakage

## Abstract

The aim of this study was to examine the effects of acupuncture on urinary incontinence and to discuss why these acupoints were selected. Seven databases were searched for any randomized controlled trials (RCTs) that investigated the use of acupuncture or acupressure as a treatment for urinary incontinence, and the Cochrane risk of bias tool was utilized to evaluate the risk of bias in each study. Four RCTs met all the inclusion criteria. The results from the selected RCTs failed to demonstrate any statistically significant improvements in urinary incontinence, although acupuncture or acupressure did exhibit favorable effects on overactive bladder symptoms and quality of life, in comparison with other conventional therapies. There have been limited results supporting acupuncture or acupressure as an effective treatment method for urinary incontinence; therefore, further RCTs are required to confirm the effectiveness of acupuncture or acupressure in the treatment of urinary incontinence.

## Introduction

Acupuncture has been performed in Korea for thousands of years and is still actively practiced in clinics today. In particular, acupuncture is known to be useful due to its pain relieving effects; however, at present, it is also studied and used for the treatment of broader symptoms, such as urinary incontinence. Urinary incontinence is a common condition, affecting ~25% of premenopausal and 40% of postmenopausal women. While it may not be a critical problem, it reduces the quality of life; however, only approximately one in four women with urinary incontinence consult a doctor about their condition (1).

Stress and urge incontinence are the two most common types of urinary incontinence, and mixed incontinence, which is a combination of the two, is also prevalent. Stress incontinence occurs when the bladder pressure exceeds urethral resistance as a result of an increased abdominal pressure, for example due to sneezing or coughing (2). Stress incontinence is defined as an involuntary leakage when the abdominal pressure increases, whereas urge incontinence occurs as a result of uncontrolled abnormal detrusor contractions that exceed the urethral pressure (2). The etiology of the urinary urgency that causes urge incontinence has not yet been fully elucidated, although some peripheral nerves, as well as the central nervous system, may be involved in muscle hypersensitivity and the reduced effectiveness of smooth muscle relaxation (3).

Acupuncture was initially described as a successful treatment for certain overactive bladder symptoms in the 1980s (4,5), and, since then, a number of clinical trials on incontinence have been carried out (6–8). There have been numerous clinical trials and some randomized controlled trials (RCTs) on urinary incontinence due to bladder instability or stress incontinence; however, the results are limited and there have been no systematic reviews to date.

The acupoints used in the studies were selected based on Korean Medicine theory, and their mechanism may be understood with reference to the autonomic and somatic nerve innervations to the bladder. Certain studies have indicated that acupuncture may influence the autonomic nerve system (9). There has not been sufficient consideration of the depth of the acupuncture insertion into the body in previous studies, and, therefore, this review aimed to provide information as to why insertion depth is necessary as a differential factor.

In this review, we examined the efficacy of acupuncture with regard to urinary incontinence, and assessed the levels of the autonomic efferent and afferent pathways and the depth of the needle insertion in the acupuncture.

There is a complex rationale behind the choice of acupuncture points for incontinence. The muscles controlling micturition are controlled by the autonomic and somatic nervous system. The detrusor muscle is relaxed by sympathetic stimulation that originates from the lumbar spinal cord T11-L2 region, and contracted by parasympathetic stimulation from the sacral spinal cord S2-4 region. The external urethral sphincter is under somatic control (10). Therefore, the coordination of the autonomic and somatic nerves to the bladder and urethra is important not only in micturition, but also in incontinence.

The points that are most commonly used in acupuncture are BL31, BL32 and BL33, which are located above the first, second and third sacral foramina, which lie over the first, second and third sacral nerve roots, respectively. These points are frequently used, due to the fact that they correspond with the segmental innervation of the parasympathetic nerve supply to the bladder.

The acupuncture points that are known to affect the micturition center and parasympathetic innervation to the urinary system include BL23, BL28 and several points on one of the eight extra meridians in the lower abdomen (11). BL23 is located at the L2 level, BL28 is located paravertebrally from the second sacral foramina and points three, four and six on the CV are located in between the pubic and the umbilical cord.

In Korean Medicine, acupoints SP6, ST36 and KI3, located on the legs, are also considered to assist bladder function by invigorating energy. Furthermore, these acupoints on the legs correspond to the skin dermatomes from L4-S2 innervation, which means that stimulation of these points may influence bladder function. Similarly, points three, four and six on the CV correspond to the skin dermatomes from T11-L1.

Since the afferent and efferent innervations of the bladder are affected by the sympathetic nerves originating at T11-L2, as well as the parasympathetic and somatic nerves originating at S2-4, these acupoints appear to be strongly organized segmentally.

In the majority of the remaining studies on bladder dysfunction, the stimulation points (not restricted to acupuncture) were selected in accordance with the segmental innervation of the nerves. Electrical nerve stimulation (TENS) applied over the pubic area, which pertains to the sacral dermatome, has exhibited certain positive effects (12), while other animal studies have also demonstrated that stimulation on the perineal area affects the bladder and sphincter function (13).

The locations of BL31, BL32 and BL33 correspond with the origin of the somatic fibers of the pudendal nerve, which provide excitatory innervation to the pelvic floor muscles and the sphincter so that they are able to remain in a contracted state. A previous study has described the beneficial effects of transcutaneous stimulation of the pupendal nerve on incontinence (14). The authors hypothesized that sacral nerve stimulation depended on the stimulation of afferent axons (which was by electrical stimulation in this particular study) in the spinal roots, and that this led to the modulation of the voiding and continence reflex pathways in the central nervous system (14).

The acupoint ST36 is predominantly used in the treatment of gastrointestinal symptoms, although it may also be used for urinary disorders. In a study investigating the ST36 acupoint, functional magnetic resonance imaging (fMRI) results indicated activation in the hypothalamus and bilateral prefrontal cortex, suggesting that acupoint ST36 activated the neural brainstem-thalamus-cortex reticular system and increased the neurotransmitter concentrations (15). It is for this reason that acupuncture has been proposed to affect micturition through the descending serotonergic system. At present, there are numerous experimental and clinical data supporting the theory that the serotonergic input facilitates glutamate-induced activation of the pudendal nerve, and thereby helps to maintain the external urethral sphincter in a closed state (8). However, there are also physiological similarities between muscle training and acupuncture, and there have been suggestions that acupuncture may be regarded as an artificial method of muscle training (16).

With regard to the insertion depth of the acupuncture needles, it has been observed that the majority of the studies concerning acupuncture have focused on the efficacy of the acupoint itself. Although electroacupuncture is frequently studied, there has been little investigation surrounding the insertion depth or the thickness of the acupuncture needle. The area of acupuncture that is predominantly focused upon is its analgesic effect on pain, and therefore most of the studies or trials concerning the depth of acupuncture have been restricted to its pain relieving effects (17,18). However, even in consideration of these studies, an appropriate penetration depth of the acupuncture needle has not been suggested. In patients with lower back pain, it has been observed that deep insertion into the muscle produced greater efficacy than shallow insertion (19). Furthermore, it has been demonstrated that, with regard to analgesic effects, the insertion of acupuncture needles into the muscles affects the nociceptors, while insertion purely into the skin does not (20). Therefore, it has been indicated that acupuncture may have different effects, according to insertion depth; however, as of yet, there is no standard regarding the depth of insertion.

While there have been relatively few clinical trials concerning incontinence that have considered the insertion depth of the acupuncture, those trials that have mentioned the depth of insertion lack consistency, as well as an explanation regarding the reason for changing the depth of insertion (8,21,22).

For the purpose of skin dermatome stimulation, the acupuncture needle has to be inserted shallowly, so that it only penetrates the skin. With regard to urinary dysfunction, the acupoints on the limbs are associated with the skin dermatomes from the spinal innervation that influence bladder function, as are the acupoints on the lower abdomen (CV 3, 4 and 6). Therefore, when acupuncture is applied to these acupoints, in order to influence bladder function, it is necessary for the depth of insertion to be no deeper than the skin. However, if the purpose is to stimulate the muscle or nerve, a deeper insertion is required. Therefore, it is necessary for the needles at the acupoints on the spinal part of the BL meridian to be inserted deeper than those at other BL points. In addition, it is necessary that the application of acupuncture to strengthen the muscle that assists the act of urination has a penetration that is sufficiently deep for the muscle to be reached.

Therefore, aside from the acupuncture point itself, there is a requirement for the insertion depth of the acupuncture needle to differ according to the intention of the stimulation.

## Materials and methods

### Data sources

The following sources were researched up to August 2012: Korean electronic databases (which included KISS, JISTI, DBPIA and the Korean Traditional Knowledge Portal), MedLine, The Cochrane Library and the Cumulative Index to Nursing and Allied Health Literature (CINAHL).

The key words used in the search for RCTs were ‘acupuncture’ AND ‘bladder’ OR ‘incontinence’ OR ‘overactive’ OR ‘urgency’ OR ‘urinary’. In Korean, Acupuncure AND (‘Urinary incontinence’ OR ‘Bladder’) were used. Each database was manually searched, independently.

#### Study selection

##### Types of studies

The review was restricted to RCTs on humans that compared acupuncture or acupressure with a control group and that included pharmacological treatment, nonpharmacological treatment, such as pelvic floor muscle exercises, sham acupuncture and placebo acupuncture (relaxation point acupuncture). Articles written in English or Korean were included with language restriction.

##### Types of participants

The review included participants of all ages who suffered from urinary incontinence. The exclusion criteria comprised individuals who had original underlying diseases or had a secondary illness, such as diabetic bladder dysfunction or a hysterectomy.

##### Types of intervention

Clinical trials evaluating acupuncture treatments, including acupressure, were included. The types of control interventions considered in this review included pharmacological treatment, nonpharmacological treatment, such as pelvic floor muscle exercises, sham acupuncture and placebo acupuncture (relaxation point acupuncture).

##### Types of outcome measure

The assessed outcomes were a reduction in the percentage of all daytime or urge accidents, in addition to changes in the Visual Analog Scale (VAS) symptom scores, self-reported severity of urine leakage and number of urine leakage episodes. The assessment of the treatments was performed using bladder diaries, the Medical Outcome Short-Form (MOS SF-36), the Incontinence Impact Questionnaire (IIQ), the Urogenital Distress Inventory (UDI), symptom questionnaires, VAS, VAS side-effects, the Psychosocial Adjustment to Illness Scale (PAIS), urodynamic assessments, cystometric tests, perineometry, four-day frequency volume charts, four-day scales, pad tests and the Chinese version of the King’s Health Questionnaire (CKHQ). The secondary outcomes assessed were the occurrence of adverse events.

##### Data extraction and assessment of the risk of bias

The full texts of the selected articles were obtained and read in full by reviewers independently. The risk of bias was assessed using the Cochrane criteria, which comprised sequence generation, allocation sequence concealment, blinding of participants, blinding of assessors, incomplete outcome data and selective outcome reporting. The assessment was based on the statement from the authors of each article (Table I).

## Results

### Study description

**Figure 1:**
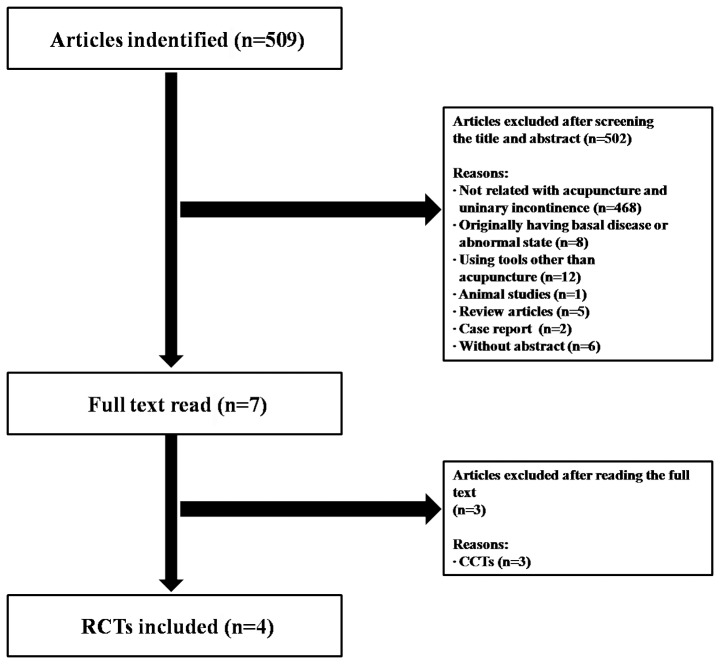
Flow chart of trial selection process. CCT, controlled clinical trial; RCT, randomized controlled trial.

The first search identified 509 potentially relevant articles, of which four met our inclusion criteria. Data extracted from the four RCTs are summarized in Table I. Trials which applied acupuncture were included. Three of the trials (8,21,22) followed a two-arm parallel group design, while one trial (23) followed a three-arm parallel group design. The trials only included manual acupuncture or acupressure, while laser- and electroacupuncture were excluded. Various acupoints for the acupuncture treatment were used in the included RCTs: SP6 was commonly selected in all four RCTs, while CV4 and BL23 were also included in two of the RCTs. Sham acupuncture treatment was conducted in three trials (20–22) and one trial (8) compared acupuncture treatment with oral medication (oxybutynin). One of the three sham acupuncture trials (23) compared combinations of acupuncture treatment and pelvic floor muscle training, sham acupuncture and pelvic floor muscle training, and pelvic floor muscle training by itself. Three of the trials (21–23) mentioned *de-qi* (the sensation of the acupuncture needle stimulating the tissue, or a feeling of numbness, heaviness or light pain that spreads around the needle) and two trials (8,21) mentioned the depth of insertion. The direction of rotation of the acupuncture needle (22) and the patients’ body posture (21) were each mentioned in one trial. Two trials originated in the USA (21,22), one in England (8) and one in Hong Kong (23). There were no statistically significant differences between the intervention and the control groups. The included studies were all in the English language and dissertations were excluded (Fig. 1).

#### Quality of Methodology

##### Sequence generation

Two studies (8,22) referred to a random number table for sequence generation. The remaining two studies (21,23) used computer-generated randomization.

##### Allocation concealment

Three (8,21,23) of the four trials received allocation scores of ‘Unclear’ as they did not provide clear descriptions of the method of allocation concealment used. The remaining trial (22) ensured that allocations were concealed with the use of sealed, opaque and sequentially numbered envelopes.

##### Blinding

The participants in one trial (8) out of the four were not blinded. The remaining studies performed participant blinding using sham acupuncture (21) or by stimulating irrelevant acupoints (22,23). The assessors of two of the trials were blinded to the group assignments, while the assessors in one of the trials (23) were not blinded and the blinding status in the remaining trial (8) was unclear.

##### Incomplete outcome data

Intention-to-treat (ITT) analysis was adopted by one trial (23). In this study, the missing outcome data were unlikely to have an effect on the true outcome, due to the fact that the withdrawals from the study were imputed from the data measured at the baseline. Two of the trials (21,22) excluded dropouts from the statistical analysis. The risk of bias in one of the trials (8) was unclear.

##### Selective outcome data

The risk of bias in the selective outcome reporting was low in three of the RCTs (8,20,21). One trial (23) used a four-point scale for evaluating the subjective severity of urine leakage; however, the results were omitted.

##### Other sources of bias

Due to the small scale of the studies, there were certain limitations to this review. The composition of the study sample was not appropriate for generalization and a larger study with a more diverse sample is required to confirm the results. For example, one study (22) did not meet the necessary number of patients to achieve an 80% power to detect a reduction in incontinence episodes. Furthermore, the participants in another study (23) were recruited from an urogynecology clinic in one acute hospital, and therefore the results may not be generalizable to other clinical settings (Table II).

#### Outcomes

##### Acupuncture versus conventional drug therapy

One RCT (8) compared acupuncture with the conventional drug treatment of 5 mg oxybutynin, twice daily. There was no significant difference in the improvement of urge incontinence between the two groups.

##### Acupuncture versus sham acupuncture

Three out of the four RCTs (21–23) compared the effects of acupuncture with those of sham acupuncture on the symptoms of incontinence. In the study by Engberg *et al*(21), the acupuncture group had a mean 67.47% reduction in all daytime urinary incontinence episodes, as compared with a mean reduction of 16.67% in the sham acupuncture group. Although the difference was not statistically significant at 4 weeks (P=0.25), it is likely that a larger sample size may yield a statistically significant difference. Similar differences were observed in the reduction of urge urinary incontinence episodes (75.20 versus 24.92%). In the study by Emmons and Otto (22), the number of incontinence episodes was reduced by 59 and 40% in the treatment and placebo groups, respectively. However, the difference between the two groups was not significant. The study by Chang *et al*(23), which used acupressure, failed to exhibit a reduction in the number of urine leakage episodes. The results failed to demonstrate statistically significant improvements in incontinence episodes, although differences were found in some of the subjective general evaluations of the treatments.

##### Adverse events

All the studies reported adverse events, although none of these were serious. However, one RCT reported discomfort due to the insertion of the needles and mild headache (8), while the other studies described drowsiness and minor bleeding (21) and bruising and discomfort with needle placement (22) (Table III).

## Discussion

This review focused on RCTs that investigated the efficacy of acupuncture or acupressure in the treatment of urinary incontinence. Several methods of sham acupuncture have been used in acupuncture RCTs, including the insertion of needles at non-acupoints (24–26), superficially puncturing the skin at non-acupoints (27–29) and the non-penetration of non-acupoints (30). Of the four RCTs selected, three trials applied sham acupuncture on the control group. One of these studies (21) used a typical sham acupuncture needle, where the needle appears to have been inserted, despite the needle not actually piercing the skin. Another study (22) selected relaxation acupoints, while the remaining study (23) used acupoints that had no relevance to urinary incontinence.

Needle stimulation causing *de-qi* has been suggested to be important for achieving the maximum effect (31,32). This sensation was considered in three (21–23) of the four selected RCTs. As mentioned previously, it has also been demonstrated that it is important for the insertion depth of the acupuncture to be varied according to the intention of the stimulation; however, none of the included RCTs reasonably differentiated the depth of acupuncture. Two trials (8,21) mentioned the depth of needle insertion: In one of the studies (8) the needle penetrated only skin-deep, while in the other study (21) the insertion depth was differentiated according the region of the body, such as whether it was the trunk or limb. It was implied that these points were selected in a manner that corresponded to the segmental innervation of the bladder, but the study did not provide a reason as to why the insertion depth was varied, i.e. deep on the trunk, and shallow on the limbs.

The majority of the included trials lacked adequate allocation concealment and involved insufficient sample sizes for meaningful conclusions to be drawn, with no inclusion of power analysis. One RCT (22) employed allocation concealment and two of the RCTs blinded the assessor. The experience and the number of the acupuncture practitioners used may be a factor affecting the results in acupuncture clinical trials. Out of the included trials, one trial (8) described the acupuncturist as ‘experienced’, while another trial (21) simply mentioned the institution that licensed the acupuncturist. In the study by Emmons and Otto (22), the acupuncture was performed by an obstetrics and gynecology doctor who had been trained in acupuncture, instead of a specifically trained acupuncturist. In one study with acupressure (23), the investigator had taken a one-month training course on acupressure theory and practice, solely for the purpose of the study. Therefore it was not fully elucidated whether the acupuncture was administered by well-trained practitioners.

All the included studies evaluated the adverse events or possible risks of acupuncture, which were either mild or not present. However, these results may not be generalizable, due to the fact that the patients with a high or a medium risk of experiencing adverse effects were excluded, the follow-up may have been short-term and the sample sizes were relatively small (33,34).

Together, the results of the evaluated studies failed to statistically demonstrate the specific effects of acupuncture on urinary incontinence. Two (21,22) of the four RCTs suggested that a reduction in the number of incontinence episodes was achieved, but did not reveal any significant differences compared with the control group. The use of statistics does not always guarantee validity, however. It is not possible to clearly determine the effect of acupuncture from the results of the four RCTs in this review.

Despite this fact, this review verified many additional effects with regard to various overactive bladder symptoms. In particular, one trial (8) revealed acupuncture to be equally efficient compared with standard anticholinergic therapy in the management of low bladder compliance, although with fewer side-effects. In addition, this study (8) demonstrated that acupuncture exhibited statistically significant effects on the symptom relief of urgency, frequency, and nocturia, as revealed on a VAS and in a voiding diary. Furthermore, there was an increase in bladder capacity and a reduction in detrusor pressure rise on filling, although there was no significant difference between the control and treatment groups in the urodynamic results. In the treatment group in another study (22), there were significant reductions in frequency, a 30% reduction in the proportion of voids correlated with urgency and a 13% increase in maximum voided volume and maximum cystometric capacity. Moreover, one of the studies (23) revealed a significant difference in the increase of pelvic floor muscle strength across three groups: intervention group (acupuncture and pelvic floor muscle training) versus sham group (sham acupuncture and pelvic floor muscle training) versus control group (pelvic floor muscle training). Following intervention, the pelvic floor muscle strength increased between the intervention and sham (U=−2.31) and between the intervention and control groups (U=−2.25). With regard to the self-reported severity of urine leakage, there were significant differences between the intervention and sham (U=−2.06) and between the intervention and control groups (U=−2.48) following intervention. In addition, the studies revealed an improvement in quality of life, as measured with numerous questionnaires. In one study, which used PAIS (8), there was an improvement in the quality of life, particularly with regard to domestic, vocational and leisure activities; however, the small number of subjects limited the value of statistical comparison. There were specific areas associated with urinary symptoms that also demonstrated several changes, as well as the general quality of life. One of the true-acupuncture groups had a greater, although nonsignificant, improvement in the urge-related distress subscale of UDI than the sham acupuncture group (21). Another study revealed that the scores on the UDI and IIQ improved in the treatment and control groups, with significant differences between the two groups for the two scores (22). In a different trial (23), the intervention group demonstrated a significant improvement in all domains of the CKHQ.

The RCTs included in this review provided insufficient evidence to enable assessment of the efficacy and safety of acupuncture or acupressure in the treatment of urinary incontinence for a number of reasons. Firstly, the results from the analysis of the RCTs were limited as a result of their methodological deficiencies. Only one study (22) demonstrated acceptable allocation concealment. The details regarding the dropouts and withdrawals were described in all trials, but it was not clearly mentioned whether they were included in the final analysis. Furthermore, in this review, the interpretation of the results was limited by the different tools used in each trial. Two studies (8,21) used a retrospective checklist for the assessment of changes in symptoms. The assessment was made before and after four to six weeks of intervention, which depended on the participants’ recall memory. Retrospective accounts of participants regarding their symptoms have been demonstrated to be unreliable in previous studies. Moreover, one trial (22) used a three-day voiding diary, which despite having been demonstrated to be nearly as accurate as a seven-day diary for studying urinary incontinence, may have been less precise.

In conclusion, although the included trials revealed that acupuncture may be beneficial to patients with urinary incontinence, there is insufficient evidence to support this conclusion, due to the methodological flaws in the studies. These included unknowns in sequence generation, concealment of allocation, blinding and outcome measures. Further large, well-designed RCTs with rigorous methods of randomization and blinding and adequate allocation concealment, in addition to validated outcome measures, are therefore required.

## Figures and Tables

**Table I:** Summary of acupuncture treatment administered in each trial.

First author (yr) [country] (ref)	Experience of acupuncturist	*De-qi*	Body posture of patients	Acupuncture point	Treatment frequency	F/U	Type of needle	Stimulation method
Engberg (2009) [USA] (20)	Not mentioned (licensed by the Commonwealth of Pennsylvania)	Considered	Lay prone or sat in massage chair	KI3, SP6, BL23, BL31, BL32, BL33	Twice a week	6 weeks, treatment; 4 weeks later	Extremity points: 32 gauge, 3 cm long disposable needles Paraspinal points: 32 gauge, 4 cm long disposable needles	Extremity points: Inserted to a depth of 20–30 mm Paraspinal points: Inserted to a depth of 30–40 mm. Needles remained in place for 25 min
Kelleher (1994) [England] (8)	Experienced	Not mentioned	Not mentioned	SP6, ST36, CV3 or 4, BL23, BL28. 2 paravertebral, lumbar, segmental points, 4 sacral, segmental points	Weekly treatments	6 weeks, treatment; 3 months later	36 gauge, 3 cm long, disposable needles	Minimal stimulation technique: Needles were merely flicked into the skin, left *in situ* a few millimetres below the skin without further stimulation for 10 min
Emmons (2005) [USA] (21)	Not mentioned (obstetrician- gynecologist)	Considered	Not mentioned	SP6, BL39, BL28, CV4	Weekly treatments	4 weeks, treatment; 2–4 weeks later	Not mentioned	Needles were placed and rotated clockwise until the patient reported the sensation of *de-qi*, then were retained without further stimulation for 20 min
Chang (2011) [Hong Kong] (22)	One-month training and practice for the trial	Considered	Not mentioned	CV3, CV4, CV6, ST36, SP6, L23, BL28, BL32	3 weekly treatments	10 weeks treatment, no f/u		

yr, year of publication; ref, reference number; F/U, follow-up.

**Table II:** Results of assessing the risk of bias.

First author (yr) [country] (ref)	Sequence generation	Allocation sequence concealment	Blinding of participants	Blinding of assessor	Incomplete outcome data	Selective outcome reporting
Engberg (2009) [USA] (20)	Y	U	Y	Y	N	Y
Kelleher (1994) [England] (8)	Y	U	N	U	U	Y
Emmons (2005) [USA] (21)	Y	Y	Y	Y	N	Y
Chang (2011) [Hongkong] (22)	Y	U	Y	N	Y	N

yr, year of publication; ref, reference number; Y, yes; U, unclear; N, no.

**Table III:** Summary of randomized controlled trials of acupuncture for the treatment of urinary incontinence.

First author (yr) [country] (ref)	N (T/C)	Population age range (mean), years	Design/blinding	Type of control	Intervention	Results	Assessment of the treatment	Adverse events (AE)
Engberg (2009) [USA] (20)	9 (4/5)	44–66 (54.8)	Parallel/SB	Sham acupuncture	Acupuncture	Percentage reduction of all daytime accident at 1 week: 63.30% vs. 18.88% NS at 4 week: 67.47% vs. 16.67% NS Urge accidents at 1 week: 74.61 vs. 16.67 NS at 4 week: 75.2 vs. 24.92 NS	7-day bladder diary (MOS SF-36) IIQ and UDI	No serious AE Drowsy (P=0.05) Minor bleeding site (P=0.05)
Kelleher (1994) [England] (8)	39 (20/19)	24–72 (51.2)	Parallel/open	Oxybutynin 5 mg twice daily	Acupuncture	Change in symptom visual analogue scores: Urge incontinence: NS	Bladder diary Symptom questionnaire VAS, PAIS Urodynamic assessment	Light headache Discomfort due to insertion of needles
Emmons (2005) [USA] (21)	74 (38/36)	22–82 (53)	Parallel/SB	Placebo acupuncture (relaxation points)	Acupuncture	Percentage change in incontinence: 59% vs. 40% NS	IIQ and UDI 3-day voiding diary Cystometric test	No significant AE. 23% bleeding, bruising. 25% minor discomfort with needle placement
Chang (2011) [Hong Kong] (22)	81 (27/27/27)	18–60 (49.57)	Parallel/SB	Sham group: Sham acupressure + pelvic floor muscle training Control group: Pelvic floor of muscle training	Acupressure	Self-reported severity of urine leakage and number of urine leakage episodes: between 3 groups: NS Self-reported severity urine leakage: intervention vs. control group: statistically significant difference (U=−2.06, P=0.04) Self-reported severity of urine leakage: intervention vs. sham group: statistically significant difference (U=−2.48, p=0.01) Number of urine leakages: NS	Perineometry 4-day frequency volume chart, 4-point scale Pad test, CKHQ	No serious AE

yr, year of publication; ref, reference number; N, number of participants; T, number of treatment group participants; C, number of control group participants; SB, single blind; NS, not significant; MOS SF-36, medical outcomes short-form; IIQ and UDI, incontinence impact questionnaire and urogenital distress inventory; VAS, visual analog scale; PAIS, psychosocial adjustment to illness scale; CKHQ, Chinese version of the King’s health questionnaire.
